# A user-friendly modified pore-solid fractal model

**DOI:** 10.1038/srep39029

**Published:** 2016-12-20

**Authors:** Dian-yuan Ding, Ying Zhao, Hao Feng, Bing-cheng Si, Robert Lee Hill

**Affiliations:** 1 State Key Laboratory of Soil Erosion and Dryland Farming on the Loess Plateau, Northwest A&F University, Yangling, Shaanxi 712100, P. R. China; 2 College of Water Resources and Architecture Engineering, Northwest A&F University, Yangling, Shaanxi 712100, P. R. China; 3 School of hydraulic energy and power engineering, Yangzhou University, Yangzhou 225127, P. R. China; 4 Xinjiang Institute of Ecology and Geography, Chinese Academy of Sciences, Urumqi, 830011, P. R. China; 5 Department of Environmental Science and Technology, University of Maryland, College Park, MD 20742, USA

## Abstract

The primary objective of this study was to evaluate a range of calculation points on water retention curves (WRC) instead of the singularity point at air-entry suction in the pore-solid fractal (PSF) model, which additionally considered the hysteresis effect based on the PSF theory. The modified pore-solid fractal (M-PSF) model was tested using 26 soil samples from Yangling on the Loess Plateau in China and 54 soil samples from the Unsaturated Soil Hydraulic Database. The derivation results showed that the M-PSF model is user-friendly and flexible for a wide range of calculation point options. This model theoretically describes the primary differences between the soil moisture desorption and the adsorption processes by the fractal dimensions. The M-PSF model demonstrated good performance particularly at the calculation points corresponding to the suctions from 100 cm to 1000 cm. Furthermore, the M-PSF model, used the fractal dimension of the particle size distribution, exhibited an accepted performance of WRC predictions for different textured soils when the suction values were ≥100 cm. To fully understand the function of hysteresis in the PSF theory, the role of allowable and accessible pores must be examined.

Soil water retention curve (WRC) is an important hydraulic parameter^1^,^2^. It is required in the simulation of unsaturated soil water movement. Various models have been developed to predict the WRC, and the models developed by Brooks and Corey (1964)^3^ and van Genuchten (1980)^4^ were commonly used.

The mathematical bases of the Campbell model^5^ (C; Eq. 1), Brooks-Corey model^3^ (BC; Eq. 2)) and van Genuchten model^4^ (VG; Eq. 3) are empirical in nature. The BC and VG models are stated in the following key equations that require the value of the water residual term to be specifically set to zero in order to obtain reasonable model predictions of the WRC.


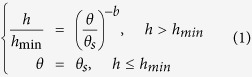



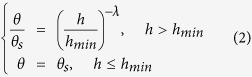



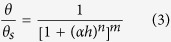


where *θ* is the water content; *h* is the suction head; *θ*_*s*_ and *h*_*min*_ are the saturated water content and the air-entry suction, respectively; and *λ, b, n*, and *m* = 1 − 1*/n* are the empirical shape parameters. The inverse of *α* is often referred to as the air-entry value. For the BC and C models, these simple equations have considerable advantages with regard to the development of closed conductivity models; however, the physical meaning of the fitting parameters used in these models is minimally interpreted^6^.

Recent use of fractal geometry concepts in soil science has resulted in the development of fractal-based models for prediction of the WRC^7^,^8^,^9^,^10^. These fractal models are useful tools that bridge the gap between the empirical models and provide some physical basis of the model parameters^11^,^12^,^13^. Tyler and Wheatcraft (1990) introduced a fractal model to predict the WRC using the Sierpinki carpet model^6^. Huang *et al*.^9^ developed a fractal model to predict the WRC using a specially constructed Menger sponge^9^. Based on pore-solid fractal (PSF) theory, the PSF WRC models proposed by Perrier *et al*.^10^ and Bird *et al*.^7^ can predict the WRC; they became collectively known as the PSF model^7^,^10^.

Unlike the conventional fractal model, the PSF model incorporates the symmetry between the solid and the pore phases. The principles are based on a self-similar local soil structure that may be described over a range of scales. This model characterizes the soil water desorption according to the soil structure fractal description and has been used to facilitate the identification of both particles and aggregated substructures over a range of scales^14^. The most commonly applied computational formulation of the PSF model is as follows:


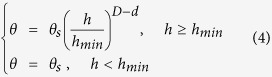


where *D* is the fractal dimension and *d* is the Euclidian dimension (i.e., *d* = 3).

This equation has a simple formula and only needs three parameters. Several methods to estimate *D* have been developed using the soil aggregate size distribution (ASD)^15^ and/or the soil particle size distribution (PSD)^7^. The saturated volumetric water content (*θ*_*s*_) may be measured; however, determining the air-entry suction (*h*_*min*_) is difficult because of the tedious measurement techniques involved^16^. Given the absence of measured values for *h*_*min*_, researchers have to initially fit Eq. (4) to the measured data before using that equation to obtain the singularity values for *h*_*min*_ and *θ*_*s*_ on the WRCs^7^,^17^. The question has emerged why only the singularity points (*h*_*min*_, *θ*_*s*_) can be used to predict WRCs. The other points on the WRC might also be able to predict the WRCs. Especially, the PSF model could directly predict the WRCs without the additional fitting progess when *D* and another calculation point on the WRCs are known.

In the current study, the PSF model was investigated using other calculation points on the WRCs to determine the entire WRC according to the fractal theory^7^. The objectives of this study were to (1) evaluate the PSF model using a range of calculation points on the WRCs using the moisture desorption and adsorption processes based on the PSF theory; (2) analyze the parameter sensitivity of this model using soil samples obtained from the Loess Plateau in China; and (3) test the effectiveness of the modified PSF model with regard to the prediction of the WRC using 54 soil samples, which represent a range of 10 soil textural groupings within the Unsaturated Soil Hydraulic Database (UNSODA).

## Theory

### Theory of the original PSF model

**Figure 1:**
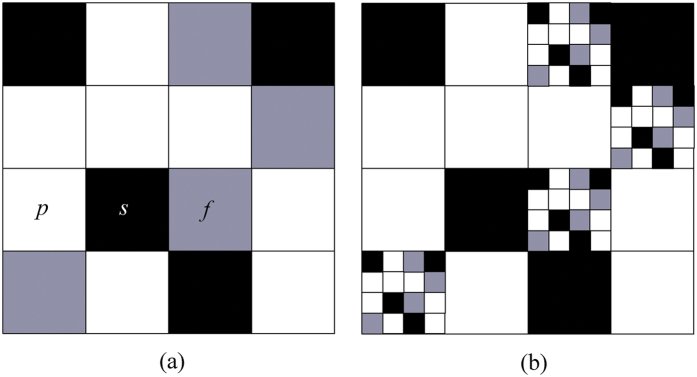
Idealized illustration of a pore solid fractal (PSF) structure. (**a**) is a subunit of the entire structure and *p* represents (white) pores, *s* represents (black) solids, and *f* (grey) represents the defined PSF aggregates, and (**b**) the idealized illustration of the subunit fitting into a larger PSF structure.

A certain volume of soil consists of the following three phases (Fig. 1a): pore (*p*), solid (*s*), and fractal (*f*)^7^. The summation of the phases represents the entire soil volume, as shown in the following equation:


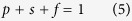


The initiator (a uniform mass fractal) in the iterative partitioning of a bound region in the Euclidean space is the description of the local structure that is similar to the entire structure (Fig. 1b, self-similarity). Introduction of the three phases could be regarded as the first stage in the construction of a fractal set in Fig. 1a. the *p* phase is assumed to be initially composed of *N*_*i*_ pores of size *r*_(*i*)_. The *s* phase is also assumed to be initially composed of *M*_*i*_ solids of size *r*_(*i*)_, which is repeated at a smaller scale for each sub-region of the *f* phase (Fig. 1b). Further iteration of this process yields a hierarchically constructed multi-scale structure^7^.

When the PSF theory distribution is used to characterize the soil texture and the iterations of its construction approaches infinity, the WRC may be directly estimated from a power function description of the PSD under the assumption that both the particle size distribution and the pore size distribution have identical fractal dimensions.

The cumulative mass distribution in three-dimensional Euclidean space may be expressed as follows^7^:


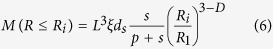


where *M*(*R* ≤ *R*_*i*_) is the total mass of the particle elements each with a size smaller or equal to *R*_*i*_, *L* is the linear size of the initiator, *ξ* is a constant and *L*^3^*ξ* is the bulk volume of the PSF, *d*_*s*_ is the particle density, *R*_1_ is the size of the largest particle element, and *D* is the fractal dimension of the cumulative mass distribution.

Based on PSF theory, a generalized PSF model for estimation of the WRC may be obtained as follows:


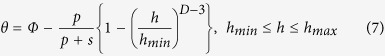


where *Φ* is the total soil porosity, and *h*_*min*_ and *h*_*max*_ are the capillary suctions that explicitly define the smallest and largest pores, respectively, within the soil structure that undergoes desaturation according to the PSF theory. Three special conditional cases have been identified where *p* = 0 and *s* *≠* 0, *p* *≠* 0 and *s* = 0, and *p* *≠* 0 and *s* *≠* 0. The result of the third conditional case is described in Eq. (4). The third case generally occurs within soils and this conditional case may serve as the basis of the future discussion. The original publication of Bird *et al*.^7^ provides additional details on the formula derivation^7^.

### Consideration of the unsaturated condition and hysteresis effect of soil

The formula derivation of Bird *et al*.^7^ describes water desorption. It starts with the biggest pore radius *r*_(1)_ (where *r*_(*i*)_ is the pore radius corresponding to the number of iterations *i, i* ≥ 0), which corresponds to the soil saturation conditions^7^. However, the soil water is generally under unsaturated condition, and this condition implies that the formula derivation of the PSF model might start with an arbitrary pore radius *r*_(*n*)_ (where *n* ≥ 0). After the description of soil fractal structure, the derivation of the PSF model can start from an arbitrary fractal iteration step that corresponds to an arbitrary pore radius *r*_(*n*)_, that is, an arbitrary point on the WRC.

Furthermore, the initial conditions of water movement may be influenced by either the moisture desorption or the moisture adsorption processes. From the viewpoint of fractal theory, the calculated fractal step (i.e., iteration step) may start at the arbitrary fractal iteration either toward a comparatively small or large fractal structure. On the one hand, the soil moisture desorption and adsorption processes may be associated with the pore-neck and pore-body size distributions, respectively, which are loosely described as the “ink-bottle” effect that contributes to hysteresis^18^. On the other hand, hysteresis is also observed during the wetting and drying of a porous medium and may be attributed to the distinction between the allowable and the accessible pores^19^. Although the two explanations of the hysteresis effect are different from each other, they both imply that the different pore size distributions (POD) must be present, and the different fractal dimensions are involved during the moisture desorption and adsorption processes. To effectively describe the soil water behavior, both the moisture desorption and the adsorption processes are considered in the derivation of the PSF model.

### Moisture desorption process

In the PSF structure, the moisture desorption process starts from the *n* time of the fractal iteration step and toward the smallest fractal structure, that is, from the pore size of *r*_*d*(*n*)_ (subscript *d* denotes the parameter in the moisture desorption process) toward the smallest pore size. *φ*_*n*+*j*_ is defined as the porosity arising from the pore size between *r*_*d*(*n*)_ and *r*_*d*(*n*+*j*)_ (where *j* is the number of iterations starting with *n, j* ≥ 0). Then, *φ*_*n*+*j*_ may be calculated as follows


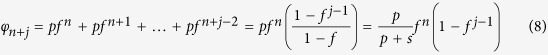


Given our assumptions that *s* > 0 and *p* > 0, Eq. (5) requires that *f* < 1. Therefore, when *j* → ∞, *f* ^*j*−1^ → 0, and 1 − *f* ^*j*−1^ → 1, the following expression is obtained:


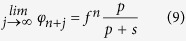


As *j* increases, the pore size decreases during the iteration process, and the water drainage synchronizes with the changing pore size. When *j* approaches infinity, all pores of size ≤*r*_*d*(*n*)_ are filled with air and *φ*_*n*+*j*_ approaches the original pore volume (*φ*_*d*_) that was filled with water at the start of desorption. According to these conditions, the following formula is obtained:


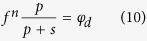


Substituting Eq. (10) into Eq. (8) yields the following:


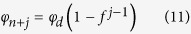


Following Bird *et al*.^7^, *f* ^*i*^ can be expressed as follows:


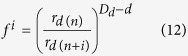


where *D*_*d*_ is the fractal dimension of the POD during the moisture desorption process, and *d* is the Euclidean dimension defined as 3. Therefore, eq. (12) may be redefined as:


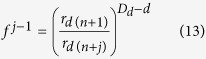


From Eqs (11) and (13), the following expression for *φ*_*n*+*j*_ may be obtained:


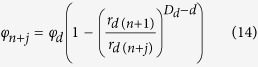


Because water desorption initially starts from the largest pores, *φ*_*n*+*j*_ can be assumed to have emptied the water retained in the defined pore size at this time. Hence, the corresponding water content *θ* is composed of water held in all pore volumes smaller than or equal to *r*_*d*(*n*+*j*)_, then


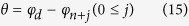


Substituting Eq. (14) into Eq. (15) results to the following expression:


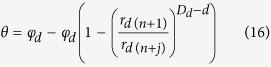


Substituting the Young-Laplace equation into Eq. (16) leads to the following expression:


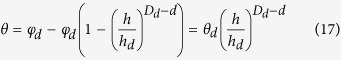


where *h*_*d*_ is the suction head at a pore size of *r*_*d*(*n*)_ and *θ*_*d*_ is the water content associated with the suction head *h*_*d*_. Note that Eq. (17) has a similar form with Eqs (1, 2 and 4).

### Moisture adsorption process

In contrast the water desorption process, the moisture adsorption process starts from the *m* times fractal iteration step to the biggest fractal structure, that is, from the pore size of *r*_*a*(*m*)_ (subscript *a* denotes the parameter in moisture adsorption) to the biggest pore size. *φ*_*m*−*i*_ is defined as the porosity arising from the pore sizes between *r*_*a*(*m*−*i*)_ and *r*_*a*(*m*)_. Therefore, the following expression is defined:


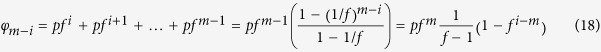


where 0 ≤ *i* ≤ *m*. Based on Eq. (12), it is determined that


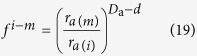


where *D*_a_ is the fractal dimension of the POD during moisture adsorption. Hence


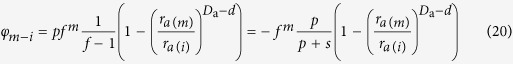


From Eqs (10) and (20),


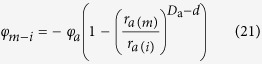


where *φ*_*a*_ is the original pore volume filled with water at the start of the water adsorption.

Substitution of the Young-Laplace equation into Eq. (21) leads to the following formula:


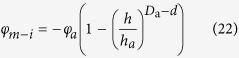


With increasing water content, the pores defined by *φ*_*m*−*i*_ are filled with water. The volumetric water content *θ* comprises of all pore volumes smaller than or equal to *r*_*a*(*m*−*i*)_, thus,


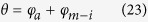


Substituting Eq. (22) into Eq. (23) results in


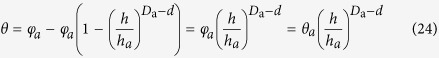


where *h*_*a*_ is the suction head for a pore of size *r*_*a*(*m*)_, and *θ*_*a*_ is the volumetric water content at the suction head of *h*_*a*_.

### Calculation of *h*_*min*_

The approach described above is different from other fractal WRC models because the singularity values for *h*_*min*_ and *θ*_*s*_ are not required in the mathematic calculation (i.e., Eqs 17 and 24). Provided that *h*_*min*_ is the same as the one value defined in the WRC, this current equation can be also applied to other fractal models, that is, the water content is assumed to have been saturated when *h* ≤ *h*_*min*_. Using the values for *θ*_*s*_ (or soil total porosity *Φ*), *D*_*d*_ (or *D*_*a*_), and one point on the WRC, for example, (*h*_*d*_, *θ*_*d*_), the *h*_*min*_ may be calculated as follows:


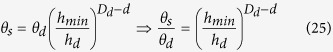


Therefore,


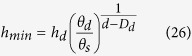


or


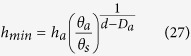


Thus, Eqs (26) and (27) indicate that the singularity value for (*h*_*min*_, *θ*_*s*_) may be calculated using another point on the WRC when the fractal dimension is known.

Based on the calculations described above, defining the complete modified PSF model (M-PSF) by combining Eqs (17, 24, 26 and 27), as follows:


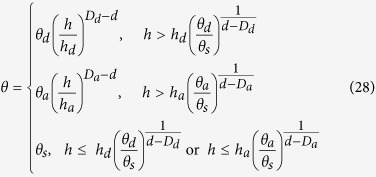


Although Eq. (28) has a form similar to that of Eqs (1 and 2) when *h* > *h*_*min*_ and has a form identical to that of Eq. (4), the model parameters are quite different and must not be considered equivalent to each other. The calculation point may be arbitrary, and *h*_*min*_ may be directly calculated using Eq. (28) rather than fitting measured data points. In Eq. (28), *D*_*d*_ and *D*_*a*_ separate from the moisture desorption and adsorption processes as stated in the fractal theory. According to the “ink-bottle” effect, *D*_*d*_ may be associated with pore-neck size distribution, whereas *D*_*a*_ may be associated with the pore-body size distribution. In addition, the main difference between the moisture desorption and the adsorption processes (i.e., the different fractal dimensions of the PODs) may be attributed to the various allowable and accessible pores in the soil wetting and drying processes.

When the WRC is estimated using a certain modeling approach, the hysteresis effect is often disregarded and only the soil moisture desorption is considered. In this case, Eq. (28) may be simplified as follows:


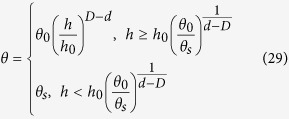


where *h*_0_ is a suction head, and *θ*_0_ is the water content at the suction head *h*_0_. In the derivation above, the fractal iteration steps *n* and *r*_(*n*)_ may be arbitrarily set, implying that *θ*_0_ and *h*_0_ may also be arbitrary (*h* > *h*_*min*_). Specifically, a pair of arbitrarily measured values *θ*_0_ and *h*_0_ on the WRC may be used as calculation points that are sufficient in the M-PSF model and thus allow the estimation of the WRC. Instead of determining specific points such as *h*_*min*_ and *θ*_*s*_ on the WRC, a large number of arbitrary points on the WRC can be effectively used in the M-PSF model.

The calculation step of the M-PSF model are summarized as follows:

1. The soil saturated water content *θ*_*s*_ or the soil total porosity *Φ* is collected (or measured).
2. Any one point on the WRC, e.g., (*h*_0_, *θ*_0_) is collected (or measured).
3. The value of *D* is calculated by the data of the ASD (or PSD).
4. The value of *h*_*min*_ is calculated by *D, h*_0_, *θ*_0_, and *θ*_*s*_ (or *Φ*) using Eq. (26). After these calculations above, the Eq. (29) can be used to estimate the WRCs.

The relationships defined as (*D* − *d*) = −λ = −1/*b* may be obtained by comparing Eq. (4) with Eq. (1) and with Eq. (2) and may explain the physical meaning of the power exponent in the BC and the C models^6^. The calculation point of *h*_*min*_ and *θ*_*s*_ in the PSF, BC, and C models are associated only with a specific point on the WRC. In contrast, the locations of the calculation point on the WRC within the M-PSF model (Eq. 29) may be numerous. Therefore, the PSF, BC, and C models may be regarded as special cases of the M-PSF model. Thus, the M-PSF model may be considered as a more generalized form of the PSF model that may be used to describe the WRC.

## Materials and Methods

### Dataset for parameter sensitivity analyses

**Figure 2:**
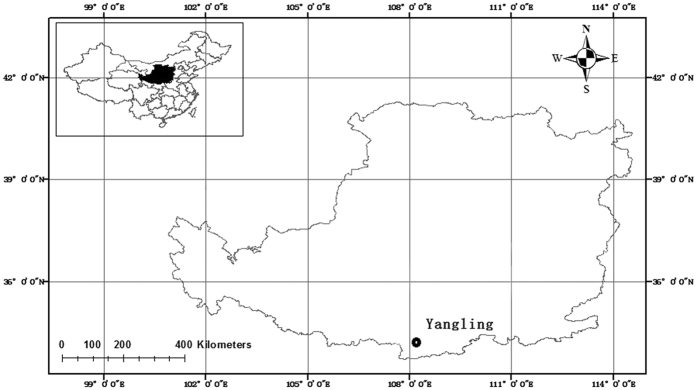
Location of Yangling on the Loess Plateau (black shading) in northwestern China. This map was created by ArcGIS 10.2 (http://www.arcgis.com/features/index.html).

A total of 26 soil samples were collected from agricultural fields at a depth of 0–10 cm in Yangling on the Loess Plateau in China (Fig. 2). For each determination, the following two associated soil samples were taken: an undisturbed core sample using 100 ml stainless steel cylinders (50 mm internal diameter and 51 mm height) with metal end caps that were used for suction and for determining the bulk density. The other samples, which were disturbed, was stored in plastic bags for aggregate and textural analyses. Soils samples from each location were analyzed in the laboratory. The aggregate mass-size distribution (ASD) was determined through the wet sieving method^20^.

The WRC was measured using a high-speed refrigerated centrifuge (HITACHI himac CR21GII), which was used to apply the desired suction head (10, 100, 300, 600, 1000, 3000, 5000, and 10000 cm). For the centrifugation method^21^, four soil samples in the cylinder samplers (100 cm^3^) were placed in a retainer of the centrifuge each time. A determined time with a constant rotation speed was used to reach the soil water potential equilibrium corresponding to a given suction head. The cylinder samplers were then removed from the centrifuge to determine their weight and soil volume. The samplers were again placed in the centrifuge to undergo a higher rotation speed. This procedure was repeated up to the last established water suction head (10000 cm). The samples were then oven-dried at 105 °C for 24 hours to obtain the soil dry mass. The soil shrinking in the cylinder sampler was offset by measuring the soil volume in each rotation speed.

The particle fractions were determined using a Malvern Mastersizer 2000. Soil textures were identified according to the US Department of Agriculture (USDA) textural classification triangle. Most of the samples were classified as silt loams, while the other samples were classified as silt clay loams. The fractional clay content ranged from 0.159 to 0.202 and had a mean of 0.183, while the fractional silt content ranged from 0.680 to 0.758 and had a mean of 0.730. The measured *θ*_*s*_ ranged from 0.435 to 0.563 cm^3^ cm^−3^ and had a mean value of 0.512 cm^3 ^cm^−3^. The calculated *D*_*A*_ ranged from 2.853 to 2.897 and had a mean value of 2.879. The bulk density ranged from 1.012 to 1.372 g cm^−3^ and had a mean value of 1.215 g cm^−3^. The fitted *h*_*min*_ ranged from 0.0042 to 2.45 cm and most *h*_*min*_ values were approximately 10 cm.

Four *h*_0_ values in the calculation point were selected, that is, 10, 100, 1000, and 10000 cm, to test the parameter sensitivity of the M-PSF model. The value of *θ*_0_ was in accordance with *h*_0_ in the measured data. *D* was calculated using Eq. (6) using ASD, which was defined as *D*_A_. When all parameters were determined, Eq. (29) can be used to estimate the WRCs.

### Dataset for testing the M-PSF model

To test the performance of the M-PSF model using the calculated *D*_*A*_, 7 soil samples with ASD data were selected from the Unsaturated Soil Hydraulic Database (UNSODA). This database contains information on the specific textures and the sample codes representing silty clay (code 3030), clay loam (code 3031, 3032, and 3033), and silt loam (3090, 3091, and 3093) soils^22^. Their calculation points were selected uniformly at *h*_0_ = 1000 cm using the corresponding *θ*_0_ values.

Moreover, a total 50 soil samples with their respective PSD data were selected from UNSODA (Table 1) to further test the performance of the M-PSF model using the PSD fractal dimension (defined as *D*_*P*_). The selected data included the WRCs determined from the undisturbed soil samples having a minimum of 10 data points over a large suction scale. Meanwhile, the associated PSD data was based on a minimum of 5 levels of particle size. The *D*_*P*_ was derived using Eq. (6). For a basic comparison, the *h*_0_ values obtained from the database ranged from 100 cm to 330 cm, and thus they were mostly 330 cm.

### Data analysis

**Figure 3:**
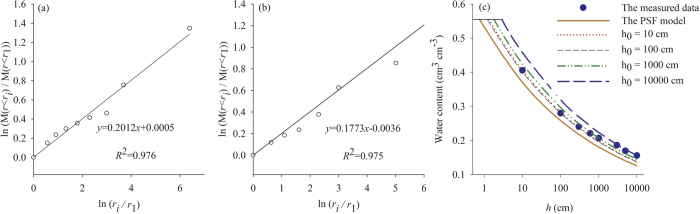
Typical examples of graphs used in the calculation of the particle size distribution (PSD) fractal dimension (*D*_*P*_), the soil aggregate size distribution (ASD) fractal dimension (*D*_*A*_), and the water retention curves (WRCs). (**a**) The logarithm of the cumulative mass of the soil particles vs. the logarithm of the upper limit of each size class of the soil particles, (**b**) the logarithm of the cumulative mass of the soil aggregates vs. the logarithm of the upper limit of each size class of the soil aggregates, and (**c**) the WRC values estimated using the pore solid fractal (PSF) model and the modified pore solid fractal (M-PSF) model using *D*_*A*_ from (**b**) as a parameter. The fitted air-entry suction value (*h*_*min*_) was 0.75 cm in the PSF model. The calculated *h*_*min*_ values used were 1.3, 1.2, 1.8 and 2.9 cm at suctions of 10, 100, 1000, and 10000 cm, respectively, for calculation points in the M-PSF model.

When Eq. (6) was used to derive the fractal dimensions, the logarithm of the cumulative mass of the soil particles was plotted against the logarithm of the upper limit of each size class (Fig. 3a). This procedure was also performed on the soil aggregate data, and the results are plotted in Fig. 3b. The fractal dimensions were obtained from the slope (3-D) of the fitted line in Fig. 3a,b. By substituting the calculated fractal dimensions and the calculation points into the M-PSF and PSF models, the WRCs were obtained (e.g., Fig. 3c). Noted that all the parameters of the M-PSF model were measured (or calculated), whereas the original PSF model, i.e., Eq. (4), had to be fitted to the measured data using the non-linear least squares fitting method to obtain the value of *h*_*min*_. In accordance with the calculation procedure of the M-PSF model in this study, the measured values of *θ*_*s*_, *D*_A_ and the fitted value of *h*_*min*_ were used in Eq. (4). Moreover, the saturated water content was reached when *h* ≤ *h*_*min*_ in the PSF model.

The root mean square errors (*RMSE*) is a measurement of the errors associated with the overestimation or the underestimation of the predicted values obtained during a simulation. The Nash-Sutcliffe coefficient (*NSE*) is a widely employed statistical tool that evaluates the goodness-of-fit of the prediction models^23^, and it has been extensively used for hydrologic model evaluations^24^. Both statistical techniques were used to test the validity of the predictions generated in the simulations using the M-PSF model. The calculations are as follows:


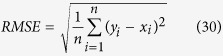



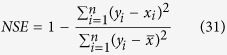


where *y*_*i*_ is the predicted value, 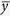 is the mean of the predicted values of *y*_*i*_, *x*_*i*_ is the measured value, 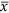 is the mean of the measured values of *x*_*i*_, and *n* is the number of samples.

## Results

### Parameter sensitivity analyses of the M-PSF

**Figure 4:**
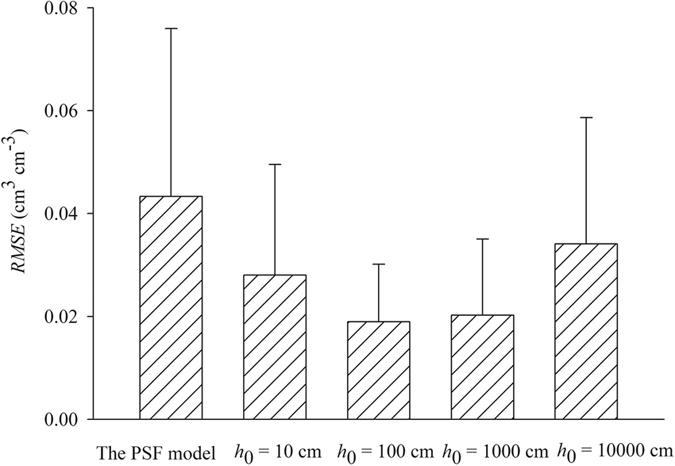
Mean values of the root mean square error (*RMSE)* between the measured and predicted water contents for the samples from Yangling using the modified pore solid fractal (M-PSF) model when the soil aggregate size distribution (ASD) fractal dimension (*D*_*A*_) was used as a parameter. The pore solid fractal (PSF) model used the fitted values of the air-entry suctions (*h*_min_) and the measured saturated water contents as calculation points. The *h*_0_ of calculation point used in the M-PSF model were selected as 10, 100, 1000, and 10000 cm. The values of the volumetric water contents (*θ*_0_) of the calculation points corresponded to the values of *h*_0_ and both the PSF and M-PSF models used *D*_*A*_ as a fractal parameter.

The evaluation results indicated that the mean values of *RMSE* of all the M-PSF model prediction levels were markedly smaller than those of the predictions obtained using the PSF model (Fig. 4). The smallest *RMSE* mean values were obtained when the M-PSF model with a calculation point at 100 cm of suction was used. Improved predictions were generally obtained using the M-PSF model when the calculation points were determined at suctions between 100 cm to 1000 cm.

The largest mean values of *NSE* were obtained when a calculation point with suction of 1000 cm was used in the calculation point, and the smallest mean values of *NSE* were observed when the PSF model was used (Table 2). *NSE* values between 0 and 1 are generally viewed as the model that demonstrates acceptable levels of performance, and values near 1 generally indicate good level of model performance^25^. *NSE* values < 0 show that the mean observed value is a better predictor compared with the simulated value. The maximum *NSE* values for both the PSF and the M-PSF models were nearly 0.99 and indicated good model performance. The minimum *NSE* value observed for the PSF model performance was negative, thus indicating an unacceptable level of model performance. The minimum *NSE* value for the M-PSF model was negative only when a calculation point of *h*_0_ = 10000 cm was used. The minimum and the maximum *NSE* values indicated acceptable levels of model performance for the M-PSF model when the calculation points at suctions between 100 cm to 1000 cm were used. When compared with the PSF model predictions, the M-PSF model performed equally well or better than the PSF model at all suction heads for the tested soils

**Figure 5:**
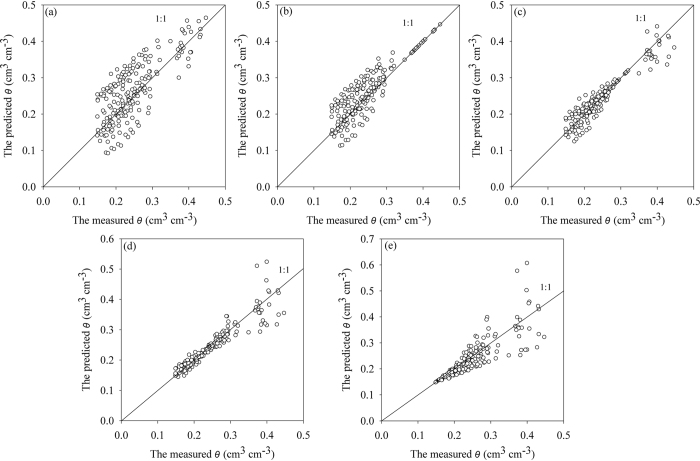
The predicated water contents vs. the measured water contents for the samples from Yangling using the modified pore solid fractal (M-PSF) model with the aggregate size distribution fractal dimension (*D*_*A*_) as a parameter. (**a**) The predictions of the pore solid fractal (PSF) model using the fitted values of the air-entry suction (*h*_*min*_) and the measured saturated water content as calculation points; (**b**) the predictions of the M-PSF model using the calculation point at a suction value of *h*_0_ = 10 cm; (**c**) the predictions of the M-PSF model using the calculation point at a suction value of *h*_0_ = 100 cm; (**d**) the predictions of the M-PSF model using the calculation point at a suction value of *h*_0_ = 1000 cm; and (**e**) the predictions of the M-PSF model using the calculation point at a suction value of *h*_0_ = 10000 cm. The volumetric water contents (*θ*_0_) of the calculation points corresponded to the values of *h*_0_ in all simulations and both the PSF and the M-PSF models used *D*_*A*_ as a parameter.

The predicted versus measured soil water contents are shown in Fig. 5. The M-PSF model with calculation points at different suctions for the topsoil samples were used. The PSF model shows an apparent tendency for dispersion as can be observed in Fig. 5a. When a calculation point at a suction of 10 cm was used, scattering was observed around water contents of 0.15 cm^3^ cm^−3^ to 0.3 cm^3^ cm^−3^. For water contents higher than 0.32 cm^3^ cm^−3^, the predicted water contents were nearly identical to the measured values (Fig. 5b). When a calculation point at a suction of 100 cm was used, the degree of scattering around the water contents from 0.1 cm^3^ cm^−3^ to 0.3 cm^3^ cm^−3^ was significantly reduced. However, the increased scattering was observed for water contents greater than 0.32 cm^3^ cm^−3^ (Fig. 5c). When a calculation point at a suction of 1000 cm was used, the predicted water contents were generally consistent with the measured soil water contents between 0.15 cm^3^ cm^−3^ to 0.3 cm^3^ cm^−3^, although the degree of scattering at water contents >0.32 cm^3^ cm^−3^ increased (Fig. 5d). Scattering also increased between the predicted and the measured soil water contents when the measured water contents were between 0.15 cm^3^ cm^−3^ to 0.4 cm^3^ cm^−3^ and a calculation point at a suction of *h* = 10000 cm was used (Fig. 5e). Generally, when the suction heads used for calibration were approaching the selected *h*_0_, improved water content estimations were obtained by comparison with the measured water contents. For all evaluations performed with the M-PSF model, the degree of scattering of the predicted values was less than the measured values when the same comparison was performed on the values predicted by the PSF model.

### Test of the M-PSF

**Figure 6:**
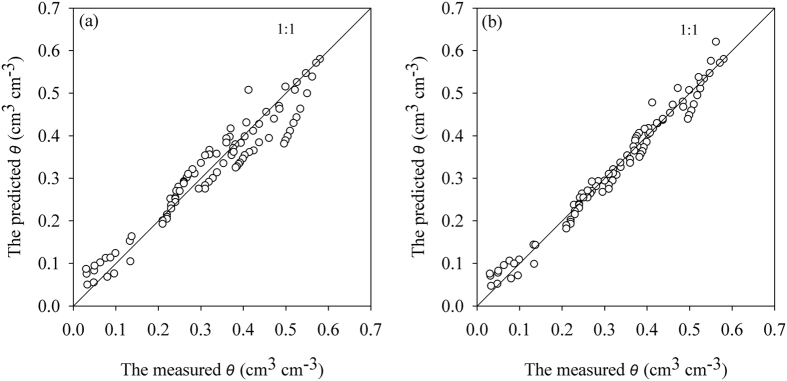
The predicted water contents vs. the measured water contents using the data from UNSODA based on modified pore solid fractal (M-PSF) model when the aggregate size distribution fractal dimension (*D*_*A*_) was used as a parameter. (**a**) The predictions of the pore solid fractal (PSF) model using the fitted values of the air-entry suctions (*h*_*min*_) and the corresponding measured saturated water contents as the calculation points; (**b**) the predictions of the M-PSF model using the calculation point at a suction of *h*_0_ = 1000 cm. Note that the volumetric water contents (*θ*_0_) of the calculation points corresponded to the values of *h*_0_ and both the PSF and the M-PSF models used *D*_*A*_ as a parameter.

The results for the Yangling soils indicated that using calculation points at suctions between 100 and 1000 cm improved the water content predictions of the M-PSF model. To verify this observation, seven samples from UNSODA were evaluated through the M-PSF model using *D*_*A*_ as the fractal component parameter and a calculation point of *h*_0_ = 1000 cm. The predicted water contents of the M-PSF model at this calculation point were consistent with the measured data, while the PSF model exhibited a higher degree of scattering (Fig. 6).

**Figure 7:**
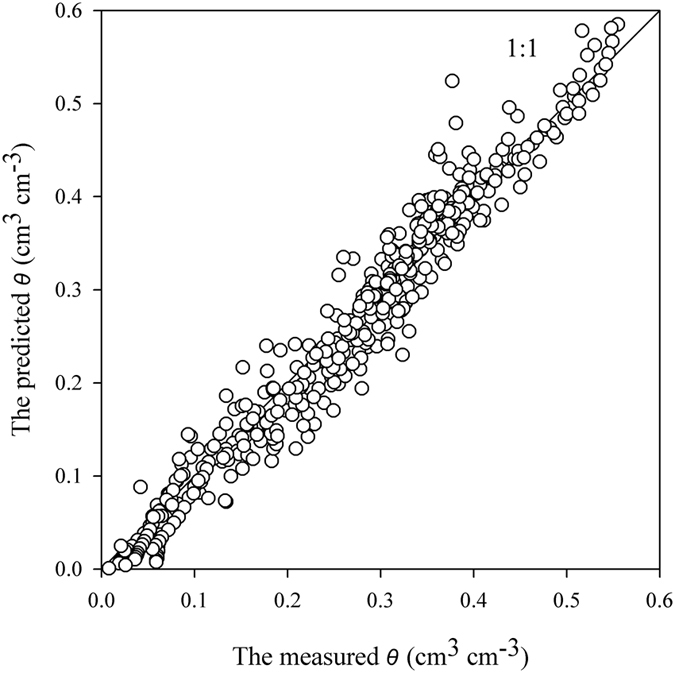
The predicated water contents vs. the measured water contents at suctions ≥100 cm using the data from the Unsaturated Soil Hydraulic Database (UNSODA) based on the modified pore solid fractal (M-PSF) model that used the particle size distribution fractal dimension (*D*_P_) as a parameter. The suction values (*h*_0_) used as calculation points of the M-PSF model were selected from the suction values that ranged from 100 to 330 cm within the database and the value that approached 330 cm was used. The volumetric water contents (*θ*_0_) of the calculation points corresponded to the values of *h*_0_ and the M-PSF model used *D*_*P*_ as a parameter.

Results of additional test using *D*_*P*_ as the soil fractal dimension indicated that the predicted water content values corresponded to the measured values when suction ranged in the values of ≥100 cm (Fig. 7). When evaluating the varied soil textures in which the suction values were ≥100 cm, the *RMSE* of the results ranged from 0.020 cm^3^ cm^−3^ for the sandy loam to 0.057 cm^3^ cm^−3^ for the clay loam (Table 3). Except the values from the clay loam soil, all *NSE* values of the results were greater than 0.5 indicating acceptable model performance. The results for the sandy loam had the largest *NSE* with a value of 0.968 (Table 3). When the suction was <100 cm, most of the water contents were overestimated. The M-PSF model generally showed good performance in predicting the values of the WRC using *D*_*P*_ as a parameter when the suction were ≥100 cm.

## Discussion

### Parameterization of the WRC according to PSF theory

The soil WRC reflects the amount of pore-filling water at different water energy states and the POD is a key characterization factor of the WRC. When the soil POD is known, an accurate estimation of the associated WRC may be obtained. The PSF theory infers values for the POD based on the symmetry of the soil pore and particle phases, and the soil particle size data may also be used to characterize the soil POD. The M-PSF model assumes that the water adsorption progresses from the smallest pores to the largest pores and that water desorption is simply the reverse process. Based on the assumption that pore desaturation at suction is inversely proportional to their sizes according to the traditional Young-Laplace equation of capillarity, the WRCs may be estimated using fractal geometry concepts. The M-PSF model starts from a general soil water state that introduces both the moisture desorption and the adsorption processes and then separately describes the two processes by using different fractal dimensions. The moisture desorption and adsorption processes use the same mathematical formulas and indicate that a power function is the appropriate mathematical function to describe both the soil structure and the POD.

### Hysteresis effect reflected by the PSF theory

The hysteresis effect observed in the soil WRCs is important because it facilitates the examination of actual water conditions in the field. In the present study, the hysteresis effect of soil is correlated with various PODs. The M-PSF model (Eq. 28) is different from traditional fractal models that neglect the hysteresis effect because it demonstrates the effects of hysteresis on the WRC by mathematically describing both the soil water adsorption and the soil water desorption processes.

Hysteresis may be caused by a change in the energy status of water when the wetting process is switched to the drying process^26^. This shift in energy status may be measured by referring to the change in the suction, that is, a Haines’ Jump^27^. The soil pore allowability and accessibility further complicate the situation^19^ because two different soil PODs in the soil wetting and drying processes are suggested and result in two fractal dimensions. The M-PSF model mathematically accounts for the change in the suction by re-estimating the POD modification involved in the adsorption and desorption processes, and the soil hysteresis effect is separately represented by the fractal dimensions: *D*_*d*_ and *D*_*a*_. In this study, we used the soil ASD or PSD to calculate the fractal dimension. The parameter *D*_*P*_ or *D*_*A*_ were regarded as the parameter *D*_*d*_ in the desorption processes with an accepted performance. However, we can not test whether or how the parameter *D*_*P*_ or *D*_*A*_ can be considered as the parameter *D*_*a*_ in the adsorption processes due to the lack of validation data. In addition, it could be possible to drive *D*_*a*_ differently, which needs a further study.

The M-PSF model directly uses the pore fractal processes that progresses from the largest to the smallest pore (or the reverse process in adsorption) that has been established in the PSF theory rather than simply using the pore draining and wetting in a process directly defined by the PODs. Similar to most fractal models, the M-PSF model disregards complicated pore networks^7^,^28^ and micro-aggregation and micro-shrinkage of soils^29^,^30^. To understand the description of the hysteresis effect comprehensively, the pore connectivity and the microscopic processes involving allowable and accessible pores should be considered in future research.

Although the M-PSF model has been theoretically formulated to consider the effects of hysteresis, this mathematical formulation requires evaluation using the WRC results for the soils that evidently demonstrate hysteresis behavior. Evaluating this behavior is beyond the objectives of this study. However, the mathematical formulation is a good first step in the theoretical consideration of hysteresis. As our understanding of the processes involved in hysteresis continues to evolve, it should be possible to build upon the mathematical formulation that has been presented.

### Comparison of the M-PSF model with other WRC models

The original PSF model requires *h*_*min*_ to be fitted similar to empirical models such as the BC and C models. Unlike these models, the M-PSF model does not require measuring of *h*_*min*_ because it may use a range of accessible WRC suction values. The most important advantage of the M-PSF model is its ability to use a wide range of calculation points. In the M-PSF derivation, the (*h*_*min*_, *θ*_*s*_) value on the WRC is replaced by a number of calculation points to make the function more practical.

The success of empirical models, such as the VG and BC models in modeling the WRC has been recognized. However, these models are strongly dependent on a large number of measured data that fit their parameters. For example, the VG model requires that measurement values equal to or greater than six must be determined^31^. When only a pair of measured data on the WRC is present, the BC and VG models cannot provide accurate estimations of the WRC. In the situation where measured data is limited, the M-PSF model can be a suitable alternative, especially when only a few measured data points from the WRC are collected. Thus, the M-PSF model is a simple and practical tool to predict the WRCs because it does not require measured data for parameter estimation.

### Selection of the parameters for M-PSF model

When the PSF models are used to predict soil WRCs, they are usually fitted to the measured data for parameter estimation^7^,^11^,^32^,^33^,^34^, In the present study, *h*_*min*_ can be calculated from the other points on the WRC using Eq. (26). *h*_*min*_ calculated by Eq. (26) was compared with the *h*_*min*_ fitted with the measured data of the 26 soil samples from Yangling. The calculated values of *h*_*min*_ ranged from 1.08 × 10^−3^ cm to 43.4 cm using a range of calculation points (i.e., 10, 100, 1000, and 10000 cm). Most of the values correspond to a suction around 10 cm, and these values exhibited the same order of magnitude as those of the fitted *h*_*min*_ values. Equation (26) provides the calculated estimations of *h*_*min*_ values that are reasonable when compared with the measured values. When the calculation points that correspond to 10 cm and 100 cm suctions are used, the calculated *h*_*min*_ values are similar to the fitted *h*_*min*_ values. Therefore, using calculation points determined at smaller suctions is recommended when using Eq. (26) to calculate the *h*_*min*_ values.

When the same fractal dimension, but different calculation points are used, several slightly different WRCs are predicted (Fig. 3c). A full range of WRC curves is obtained, and this range may be partially attributed to some aspects of the inconsecutive fractal structure of the soil POD^33^. Every textured soil may have a “most appropriate” suction range of calculation points to predict the WRC using the M-PSF model. In this study, the M-PSF model using the calculation points at suctions between 100 and 1000 cm has exhibited better performance in silt loams and silt clay loams. These soils were in the loam soil textural classification. The selection of the calculation points for clay and sand textured soils may be different, and thus it requires further determination.

Regarding the soil fractal dimensions, a considerable number of scientific journal articles have focused on the determining *D*_*A*_^35^,^36^,^37^, *D*_*P*_^38^, and soil surface fractal dimension^39^. In the present study, the M-PSF model is evaluated using both *D*_*A*_ and *D*_*P*_parameters. Some aspects of the M-PSF model performance requires further study, for example, the amount of scattering in Fig. 5 using *D*_*A*_ and the performance of M-PSF when the suction ranging in the values of <100 cm using *D*_*P*_. Furthermore, these results indicated that both *D*_*A*_ and *D*_*P*_ may be employed equally well to predict the WRCs when the M-PSF model was used. However, identifying the best fractal dimension for the M-PSF model for a range of soil textures and structures is beyond the scope of the current study. Nonetheless, this logical topic should be the focus of future studies.

### Implications from the current study

In agricultural water management, the field capacity of the soil is frequently used in irrigation planning although determining the field capacity of the soil is slow and costly^40^. Pressure-based methods define soil field capacity as the amount of water available that corresponds to a specific matric potential value ranging from 100 cm to 330 cm^41^. The permanent wilting coefficient is defined as the water retained by the soil at a soil water suction of 15000 cm. When the WRC is known, the coefficients may be easily estimated. However, the direct measurement of the WRC is costly and time-consuming because of the inherent temporal and spatial variability of the soil physical properties^42^. By using the M-PSF model, the WRC may be easily obtained when the information concerning the ASD or PSD data (i.e., the fractal dimension) and a relatively less soil water content at corresponding suctions are known. After the field capacity and the permanent wilting coefficient are estimated, a water-efficient irrigation procedure and schedule may be implemented.

Several important issues were not addressed in the current investigation. In the first instance, Ghanbarian and Daigle (2015) recently focused on the calculation of the particle-size distribution fractal dimension and stated that the lower and upper cutoffs of the fractal scaling should be considered when calculating the fractal dimension^43^. However, the PSF theory is based on the symmetry of the soil pore phase and particle phase. If the fractal dimension in Ghanbarian and Daigle (2015) was used in the M-PSF model, the entire soil POD would seem to be only associated with a small PSD range^43^, which is inconsistent with the current PSF theory. Rather than insisting the use of an entire PSD range to estimate the fractal dimension, the fractal dimension deduced from the lower and upper cutoffs of the PSD should correspond to the POD and the WRC ranges.

In the second instance, when the PSF models are used, discontinuity is observed when the soil is near saturation, thereby implying that the WRC does not follow the commonly used power function in this range or somehow changes the soil fractal dimension because of the water retained within this range. The M-PSF model, which uses the Young-Laplace equation to evaluate the matric potential, also does not consider how the fractal dimension might be sectionalized in this range of the WRC.

Addressing these two issues mentioned above may be possible by conducting additional studies to evaluate the use of piecewise fractal models, such as the models of Millán and González-Posada (2005)^33^ and/or Ojeda *et al*.^34^, and/or a variation of PSF model using a piecewise PSD (Huang and Zhang, 2005)^44^. These issues and their solutions are promising areas for additional research.

The PSF theory only describes a static system and does not consider the solid surface adsorption dynamics, energy variations^32^, and/or soil compaction issues^45^. At present, the PSF theory has not reached a point of resolution by comparison with other theories such as hydrodynamics, field theory, and potential energy theory. Because of the inherent temporal and spatial variability of soil physiochemical properties, neither a separate theory, nor a static system can effectively describe the complex water movement processes in soils. Therefore, the PSF theory can be promoted taking the solid surface suction, water potential energy, and the soil pore compaction into account in the next work. Moreover, technologies involving the original SEM^39^, X-ray^46^,^47^, and the nuclear magnetic resonance^48^,^49^ methodologies can be utilized on the soil fractal dimension using a microscopic view, which may give a solution to further determine the parameter *D*_*a*_ and *D*_*d*_ in future studies.

In conclusion, this study presented a modified PSF model (M-PSF) to easily simulate the WRC and theoretically evaluate the effects of hysteresis. The M-PSF model differs from conventional PSF models because its calculation points are numerous and can be arbitrarily chosen on WRCs. Thus, the M-PSF model is more accessible than the previous PSF models and can provide an extensive range of alternatives for parameter selection.

## Additional Information

**How to cite this article**: Ding, D.-Y. *et al*. A user-friendly modified pore-solid fractal model. *Sci. Rep.* **6**, 39029; doi: 10.1038/srep39029 (2016).

**Publisher's note:** Springer Nature remains neutral with regard to jurisdictional claims in published maps and institutional affiliations.

## Figures and Tables

**Table 1:** The soil textural classifications and selected Unsaturated Soil Hydraulic Database (UNSODA) codes for the soil particle size distribution data.

Texture	Number of data sets	UNSODA codes
Clay	5	2360, 2361, 2362, 4680, 4681
Clay loam	4	1123, 3031, 3032, 3033
Loam	11	1370, 2530, 2531, 3190, 3191, 3192, 3193, 3194, 3195, 3221, 3222
Silt loam	14	1280, 1281, 1490, 2001, 2002, 2010, 2011, 2012, 3360, 3361, 4510, 4530, 4672, 4673
Silt	1	4670
Silty clay loam	4	1164, 1165, 1371, 1372
Sand	5	3340, 4650, 4651, 4660, 4661
Sandy clay loam	2	4602, 4621
Sandy loam	4	1131, 1130, 1381, 2532

**Table 2:** The Nash-Sutcliffe coefficient (*NSE)* values evaluating model performance in the prediction of the water content for the soil samples from Yangling using the modified pore solid fractal (M-PSF) model when the aggregate size distribution fractal dimension (*D*_*A*_) was used as a parameter.

Situations	*NSE*		
	Max	Min	Mean
The PSF model	0.995	−1.621	0.344
*h*_0_ = 10 cm	0.995	0.034	0.736
*h*_0_ = 100 cm	0.994	0.503	0.893
*h*_0_ = 1000 cm	0.997	0.084	0.859
*h*_0_ = 10000 cm	0.994	−1.219	0.599

The pore solid fractal (PSF) model used the fitted values of the air entry suction (*h*_*min*_) and the measured saturated water content as calculation points. The values of suction (*h*_0_) used as calculation points for the M-PSF model were selected as 10, 100, 1000, and 10000 cm, respectively. The values of the volumetric water content (*θ*_0_) at the calculation points corresponded to the values of *h*_0_ and both the PSF and M-PSF models used the *D*_*A*_ as a parameter.

**Table 3:** The root mean square errors (*RMSE)* and the Nash-Sutcliff (*NSE)* values evaluating model performance for the results of the modified pore solid fractal (M-PSF) model for the soils data from the Unsaturated Soil Hydraulic Database (UNSODA).

Texture	*RMSE* (cm^3^ cm^−3^)	*NSE*
Clay	0.022	0.911
Clay loam	0.057	−0.289
Loam	0.023	0.921
Silt loam	0.034	0.887
Silt	0.052	0.861
Silty clay loam	0.039	0.754
Sand	0.023	0.689
Sandy clay loam	0.042	0.857
Sandy loam	0.02	0.968

Suctions range from values of ≥100 cm and the fractal dimension was calculated from the particle size distribution (*D*_*P*_). Here the values of *h*_0_ in calculation points of M-PSF model were selected the values close to 330 cm (ranging from 100 to 330 cm). The values of the volumetric water content (*θ*_0)_ of the calculation points corresponded to the values of *h*_0_.
